# Combining Machine Learning and Molecular Dynamics to Predict Mechanical Properties and Microstructural Evolution of FeNiCrCoCu High-Entropy Alloys

**DOI:** 10.3390/nano13060968

**Published:** 2023-03-07

**Authors:** Jingui Yu, Faping Yu, Qiang Fu, Gang Zhao, Caiyun Gong, Mingchao Wang, Qiaoxin Zhang

**Affiliations:** 1 School of Mechanical and Electronic Engineering, Wuhan University of Technology, Wuhan 430070, China; 2 Hubei Key Laboratory of Mechanical Transmission and Manufacturing Engineering, Wuhan University of Science and Technology, Wuhan 430081, China; 3 Wuhan Institute of Marine Electric Propulsion, Wuhan 430064, China; 4 Centre for Theoretical and Computational Molecular Science, Australian Institute for Bioengineering and Nanotechnology, The University of Queensland, St Lucia, QLD 4072, Australia

**Keywords:** high entropy alloy, machine learning, molecular dynamics, mechanical properties

## Abstract

Compared with traditional alloys, high-entropy alloys have better mechanical properties and corrosion resistance. However, their mechanical properties and microstructural evolution behavior are unclear due to their complex composition. Machine learning has powerful data processing and analysis capabilities, that provides technical advantages for in-depth study of the mechanical properties of high-entropy alloys. Thus, we combined machine learning and molecular dynamics to predict the mechanical properties of FeNiCrCoCu high-entropy alloys. The optimal multiple linear regression machine learning algorithm predicts that the optimal composition is Fe_33_Ni_32_Cr_11_Co_11_Cu_13_ high-entropy alloy, with a tensile strength of 28.25 GPa. Furthermore, molecular dynamics is used to verify the predicted mechanical properties of high-entropy alloys, and it is found that the error between the tensile strength predicted by machine learning and the tensile strength obtained by molecular dynamics simulation is within 0.5%. Moreover, the tensile-compression asymmetry of Fe_33_Ni_32_Cr_11_Co_11_Cu_13_ high-entropy alloy increased with the increase of temperature and Cu content and the decrease of Fe content. This is due to the increase in stress caused by twinning during compression and the decrease in stress due to dislocation slip during stretching. Interestingly, high-entropy alloy coatings reduce the tensile-compression asymmetry of nickel; this is attributed to the reduced influence of dislocations and twinning at the interface between the high-entropy alloy and the nickel matrix.

## 1. Introduction

High entropy alloys (HEAs) have the potential to be widely used in aero engines due to their excellent mechanical properties, such as high yield strength [1], high temperature oxidation resistance [2,3], low temperature ductility [4,5] and corrosion resistance [6]. Walter et al. [7] studied the hardness and compressive yield strength of as cast HfTaNbTiZr HEAs. Tang et al. [8] showed that the nano-twin behavior of Al_0.5_CoCrCuFeNi HEAs resulted in a high fatigue strength that was superior to many industrial alloys. Licavoli et al. [9] studied fracture characteristics and microstructures of two single-phase face centered cubic (FCC) HEAs, CoCrFeNi and CoCrFeNiMn. Hemphill et al. [10] prepared Al_0.5_CoCrCuFeNi HEAs using a vacuum induction furnace and studied their fatigue behavior. They found that the reduction of microstructural defects such as alumina inclusions and microcracks increased the fatigue strength of HEAs. Keli et al. [11] studied strength, ductility and fracture toughness of single-phase FCC HEA CrMnFeCoNi at room temperature. Li et al. [1] studied the volume effect in HEAs as a function of hardness and strength. Mohsen et al. [12] determined the fracture toughness and fatigue crack propagation behavior of two vacuum arc-cast Al_0.2_CrFeNiTi_0.2_ and AlCrFeNi_0.2_Cu HEAs, and revealed that fatigue strength of the HEA decreased significantly with an increase of load ratio. Fu et al. [3] studied a novel single-phase Co_25_Ni_25_Fe_25_Al_7.5_Cu_17.5_ HEA prepared by mechanical alloying and discharge plasma sintering and found that its high yield strength and hardness were attributed to the strengthening of grain boundary and dislocation.

In the experimental method to study the mechanical properties of HEA, Senkov et al. [13] showed that high compression yield strength of Ta_20_Nb_20_Hf_20_Zr_20_Ti_20_ HEA is caused by solution strengthening. Cao et al. [14] prepared a body centered cubic (BCC) structure Ti_29_Zr_24_Nb_23_Hf_24_ HEA and found that the yield stress of the HEA increased significantly with an increase of strain rate due to dislocation slipping. Tsai et al. [15] studied the effect of temperature on the mechanical properties of Al_0.5_CoCrCuFeNi HEA by mechanical alloying and rolling processing. Sun et al. [16] predicted phase formation of HEAs through thermodynamic calculations and found that the solid solution formed promoted the excellent mechanical properties of the alloy. Wu et al. [17] studied the fracture behavior of Hf_25_Nb_25_Ti_25_Zr_25_ HEA with work hardening effect caused by dislocation motion and proliferation. Li et al. [18] showed that the high strength and ductility of metastable biphasic Fe_20_Mn_20_Ni_20_Co_20_Cr_20_ HEA is attributed to the two-phase (BCC/FCC) structural transformation. Huang et al. [19] explored ductility and work hardening capabilities induced by phase change of brittle HEAs in metastable engineering. Zhang et al. [20] studied the mechanism of plastic deformation and magnetization properties of HEAs. Palguna et al. [21] revealed the high temperature deformation mechanism of Al_0.2_CoCrFeNiMo_0.5_ HEAs in the fully annealed state. They found that dynamic strain aging is the main mechanism of serrated flow. Brechtl et al. [22] revealed the mesoscopic complexity of macroscopic smooth plastic flow of Al_0.3_CoCrFeNi alloy. The visualization of the local strain rate field reveals different microscopic characteristics between smooth flow and abrupt flow. Tirunilai et al. [23] found that the key parameters for low-temperature jagged plastic deformation are temperature and dislocation density. The strengthening mechanism of superimposed faults in jagged plastic deformation was revealed. Meanwhile, in the simulation study of high entropy alloys, Lu et al. [24] reproduced the phase transition of the cluster structure from BCC to FCC during the annealing process of AlCoCrCuFeNi HEA by molecular dynamics (MD) simulation. Tian et al. [25] studied the deformation mechanism of nano-twin FeNiCrCoCu HEA and single crystal FeNiCrCoCu HEA. The difference in the deformation mechanism between the two lies in the form of dislocation nuclei and stacking faults. Qi et al. [26] studied twin-controlled inelastic deformation of FeCoNiCrMn HEAs at low temperatures and high strain rates through MD simulation.

However, experimental methods require significant cost and time to study the mechanical properties of HEAs, and it is challenging to establish mathematical models of composition ratios and mechanical properties. Machine learning (ML) is widely used to predict the mechanical property of alloys due to its good data processing and learning capabilities. Brun et al. [27] estimated the creep breaking strength of bainite and martensitic steels using neural network models and the chemical composition, heat treatment and temperature and time functions of heat-resistant steel. Conduit et al. [28] developed an artificial intelligence tool to predict mechanical properties of molybdenum-based alloys. Mohammed et al. [29] used a multilayer feed-forward topology algorithm and the backpropagation algorithm containing two hidden layers to predict the effect of chemical composition on the toughness and hardness of alloyed pipeline steel. Zhang et al. [30] found that the kernel-based extreme learning model was superior to other models in predicting yield stress and Young’s modulus. Li et al. [31] obtained CrCoFeNi HEA with high yield strength using ML and MD simulation. Zheng et al. [32] designed Ni_32_Co_28_Fe_28_Cr_3_Al_3_Ti_6_ HEAs through ML and pre-strain aging and found that generation of nanoprecipitates in the HEA resulted in excellent strength and ductility. Uttam et al. [33] used a random forest algorithm to predict yield strength of MoNbTaTiW and HfMoNbTaTiZr HEAs, which were consistent with experimental results. Qian et al. [34] studied mechanical properties of unequal atomic ratio HEAs FeCrNiCoMn and further verified the accuracy of the ML model by polycrystalline FeCrNiCoMn.

In this paper, the mechanical properties of FeNiCrCoCu HEA with different atomic ratios are predicted using ML and the optimal principal-element ratio is determined. Then, the tension-compression asymmetry of HEA at different temperatures and components was studied using the MD method. The tension-compression asymmetry of HEAs was revealed through microstructural evolution. To reveal the strengthening effect of HEA on nickel, the influence of HEA coating on the mechanical property of Ni matrix and its microscopic mechanism were further explored.

## 2. Models and Methods

**Figure 1:**
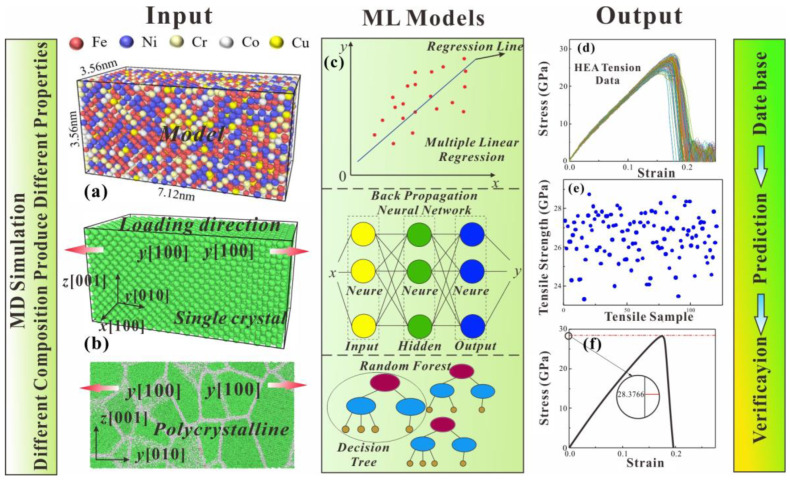
(**a**) Tensile model diagram of FeNiCrCoCu HEA, (**b**) Single crystal model diagram of HEA, (**c**) Three ML models: multiple linear regression (MLR), back propagation neural network (BPNN) and random forest (RF), (**d**) Stress-strain curves for 120 tensile models, (**e**) Tensile strength distribution of 120 tensile models, (**f**) Stress-strain curves of HEAs simulated by MD to verify optimal mechanical properties.

LAMMPS [35] (Large-scale Atomic/Molecular Massively Parallel Simulator) is used to perform MD simulations. In Figure 1a,b, the model size of FeNiCrCoCu HEA is 3.56 × 7.12 × 3.56 nm^3^ with an FCC phase structure [36]. The HEA model first relaxed at 300 K under NVT ensemble for 300 ps. The x (100) and y (010) directions of models is set to periodic boundary conditions, and z (001) direction is set to contractionary boundary condition. The strain rate of tensile and compression is 4 × 10^−7^ s^−1^ with a time step of 0.001 ps. The tensile load is applied in the y direction. The EAM [37] potential function is used to study the mechanical properties of HEAs as follows:

$$E_{i}=F_{\alpha }\underset{j\neq i}{\sum }\rho_{\beta }\left(r_{ij}\right)+\frac{1}{2}\underset{j\neq i}{\sum }\phi_{\alpha \beta }\left(r_{ij}\right)$$

where α, β are the atomic types of atoms *i* and *j*. *ϕ* is the two-body potential, which is a function of atomic type α, β and atomic distance *r_ij_*. *F* is the multi-body potential, which is equal to the potential energy generated by the interaction of electron density contributed by other atoms *j* at atom *i*. Electron density ρ provided by atom *j* to atom *i* is only a function of atomic type of *j* and atomic spacing *r_ij_*. This reflects the isotropic characteristics of metal bonds. After testing, it is verified that this potential function can adequately simulate various microstructures in FeNiCoCrCu HEA. Short-range ordering has a significant impact on mechanical properties [38]. The EAM parameters are shown in Table 1.

To fully consider the effect of the content of the principal component on the tensile and compressive mechanical properties of HEA, the five atoms of FeNiCrCoCu are randomly distributed. OVITO [39] (open visualization tool) was used to analyze the microstructural evolution of HEAs under tensile and compressive loads. Microstructural evolution features were extracted by microscopic characterization methods such as centro-symmetry parameter (CSP) [40], common neighbor analysis (CNA) [41] and dislocation analysis (DXA) [42]. Multiple linear regression (MLR) [43], back propagation neural network (BPNN) [44] and random forest (RF) [45] models were used to predict the mechanical properties of FeNiCrCoCu HEAs in Figure 1c. The number of decision trees selected by the RF model is 20, and the BPNN model uses the gradient descent algorithm, in which the set hidden layer is 10. The proportion of the primary elements of FeNiCrCoCu HEAs is used as input, and tensile strength is used as output for mechanical learning. HEA components are designed with a minimum input limit of 5% and an upper limit of 35% in ML.

## 3. Results and Discussion

### 3.1. Effect of Principal Element Content on Mechanical Properties of HEAs

**Figure 2:**
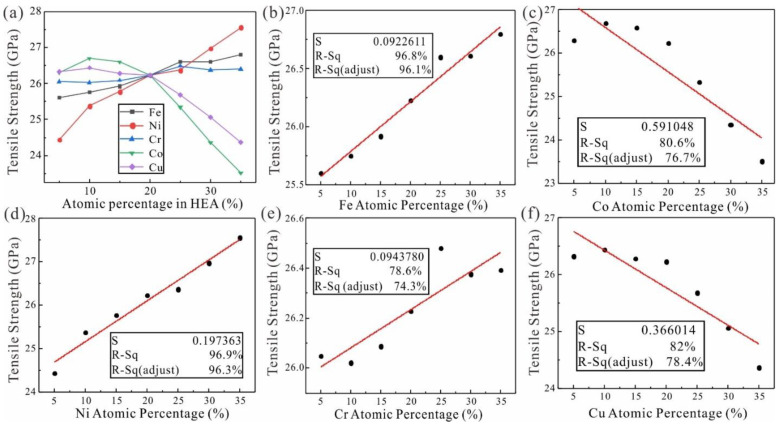
(**a**) Linear correlation between tensile strength of principal elements of FeNiCrCoCu HEAs with unequal atomic ratio, (**b**) Fe, (**c**) Ni, (**d**) Cr, (**e**) Co, (**f**) Cu and FeNiCrCoCu HEAs. S, R-Sq and R-Sq (adjust) represent the standard error, the goodness fit and the goodness fit adjusted for multiple parameters, respectively.

To determine the relationship between the tensile strength of HEA and the content of principal elements, MD simulation was carried out for the tension of FeNiCrCoCu HEA models with different contents. The linear goodness-of-fit R-Sq between tensile strength of FeNiCrCoCu HEA and content of each principal element are relatively high in Figure 2a. R-Sq of Fe and Ni both exceeded 96%, and R-Sq of three elements of Cr, Co and Cu reached almost 80%. Therefore, there is a strong linear correlation between principal element content and tensile strength of FeNiCrCoCu HEA. With increase of the Ni content, tensile strength of HEA increases significantly in Figure 2b–f. Meanwhile, the tensile strength of FeNiCrCoCu HEA also showed an increasing trend as the increasing of Fe and Cr elements. However, the tensile strength of FeNiCrCoCu HEA first increases slowly and then decreases rapidly when the Co and Cu elements continue to increase (discussed below).

### 3.2. ML Prediction of the Component Ratio for Optimal Mechanical Property

**Figure 3:**
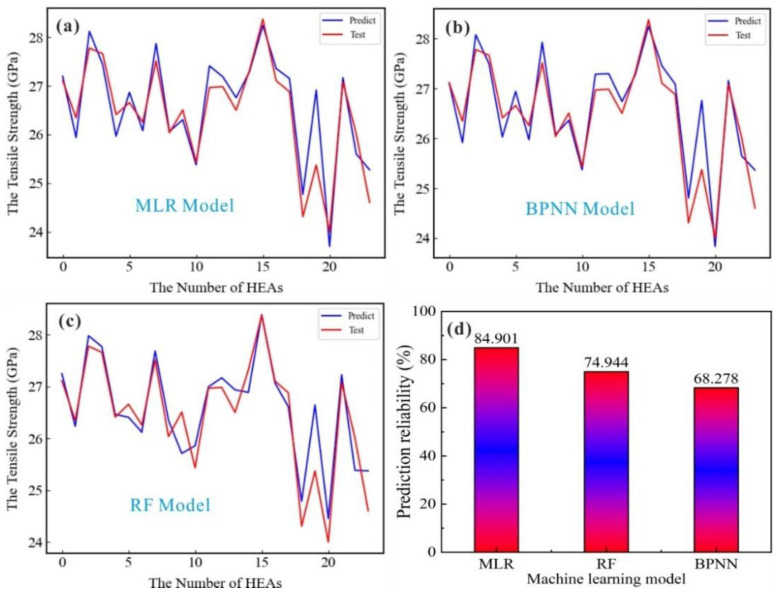
(**a**) MLR model test set prediction control; (**b**) BPNN model test set prediction control; (**c**) RF model test set prediction control; (**d**) Prediction confidence of three ML models.

The primary element content and tensile strength of FeNiCrCoCu HEA are used as input and output. Three ML models were used to predict mechanical properties; 80% of the input volume is used as the training set for ML model learning, and another 20% is used as the test set to measure the learning efficiency and accuracy of the ML model. Figure 3 shows the learning results and test accuracy of the three ML models.

Through the comparative analysis of training results of three ML algorithm models, we found that the MLR model has obvious advantages. In Figure 3, the three ML models have high confidence in the learning prediction process. The prediction confidence of the BPNN, RF and MLR models is 68.28%, 74.94% and 84.9%, respectively. The MLR model with linear prediction bias has the highest prediction confidence. Therefore, there is a strong linear relationship between the principal element content of FeNiCrCoCu HEAs with unequal atomic ratio and its tensile strength. We chose the MLR model to predict unequal atomic ratio FeNiCrCoCu HEAs with optimal tensile strength. The MLR model was re-learned and tested again, and Fe_33_Ni_32_Cr_11_Co_11_Cu_13_ HEA was predicted to have the atomic ratio with the best tensile strength.

**Figure 4:**
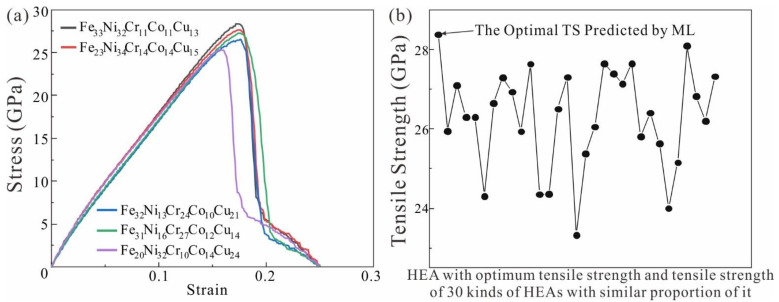
(**a**) Tensile stress-strain curves of Fe_33_Ni_32_Cr_11_Co_11_Cu_13_ HEA and HEAs with similar component proportions; (**b**) Comparison of optimal mechanical properties of HEA and HEAs with other component ratios.

To verify the accuracy of prediction, the mechanical properties of Fe_33_Ni_32_Cr_11_Co_11_Cu_13_ HEA and the HEAs with similar optimal component proportions were compared in Figure 4a. The MLR model predicted that the tensile strength of Fe_33_Ni_32_Cr_11_Co_11_Cu_13_ HEA was 28.25 GPa, while the actual simulated tensile strength of the HEA was 28.38 GPa. The error between the predicted and actual value (MD simulation) is within 0.5%, indicating that the results predicted by MLR are consistent with the actual results. To further verify the accuracy of machine learning prediction results, we selected 30 kinds of high entropy alloys with similar composition to the prediction results for simulation. The tensile strength distribution diagram for the above can be seen in Figure 4b. The tensile strength distribution map of Fe_33_Ni_32_Cr_11_Co_11_Cu_13_ HEA and HEAs with similar component proportions was obtained. The predicted elemental ratio achieves optimal tensile strength of the HEA. In Figure 4b, the tensile strength distribution map of Fe_33_Ni_32_Cr_11_Co_11_Cu_13_ HEA and HEAs with similar component proportions was obtained. The predicted elemental ratio achieved the optimal tensile strength of the HEA. From the HEA results predicted by the MLR model, the prediction results are consistent with the conclusions of the above analysis. The higher the atomic content of Fe and Ni, the higher the tensile strength of the HEA.

### 3.3. Tension-Compression Asymmetry of FeNiCrCoCu HEAs

To further study effects of temperature and principal element content on mechanical properties, tensile and compressive mechanical properties of FeNiCrCoCu HEAs at different temperatures and composition ratios were simulated. We further simulate the tension-compression asymmetry of different HEA models near the optimal element ratio in Table 2.

**Figure 5:**
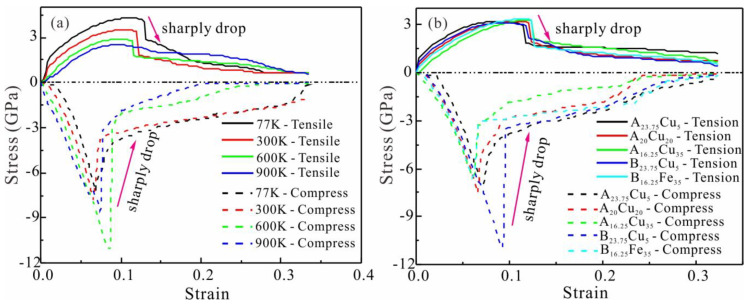
(**a**) Stress-strain curves of FeNiCrCoCu HEAs with unequal atomic ratio under tensile and compressive loads at different temperatures. (**b**) Stress-strain curves of FeNiCrCoCu HEAs with unequal atomic ratio under different component and proportions, where A*_x_* is Fe_x_Ni*_x_*Co*_x_*Cr*_x_* (*x* = 23.75, 20, 16.25) and B*_y_* is Ni*_y_*Co*_y_*Cr*_y_*Cu*_y_* (*y* = 23.75, 16.25).

Mechanical properties of FeNiCrCoCu HEAs are quite different under tensile and compressive loads at temperatures of 77 K, 300 K, 600 K and 900 K, as seen in Figure 5a. HEA has the highest tensile strength at 77 K. We found that the higher the temperature, the lower the tensile strength of HEAs. HEA drops sharply after reaching maximum stress. However, as the temperature rises, the degree of sharp stress decreases. Meanwhile, the compressive strength at the same temperature is much greater than the tensile strength, and the stress decreases faster after yielding. The tensile strength of HEAs decreases gradually as Cu increases at 300 K in Figure 5b, but its effect is much smaller than that of temperature. However, the compressive strength of HEAs increases with increasing Cu content. The effect of Fe content on tensile and compressive strength of HEA is completely opposite to the effect of Cu content.

Tension-compression asymmetry of HEAs at different temperatures varies significantly, as seen in Table 3. At 77K and 600K, the tensile-compression asymmetry of HEA was 40.5% and 73.94%, respectively. However, the content of Fe and Cu has little effect on the tensile-compression asymmetry of HEA. When the Fe content increased from 5% to 35%, the tension-compression asymmetry of HEA decreased by 22.2%. When the Cu content changed in the same range, the tension-compression asymmetry of HEA was only reduced by 7.68%. Therefore, the influence of temperature on the tension-compression asymmetry of HEA is greater than that of the main element content, and the influence of Fe content is greater than that of Cu content. To further reveal the above phenomenon, we analyzed the microstructural evolution behavior of HEA.

### 3.4. Microstructural Evolution Mechanism of Tension-Compression Asymmetry at Different Temperatures

**Figure 6:**
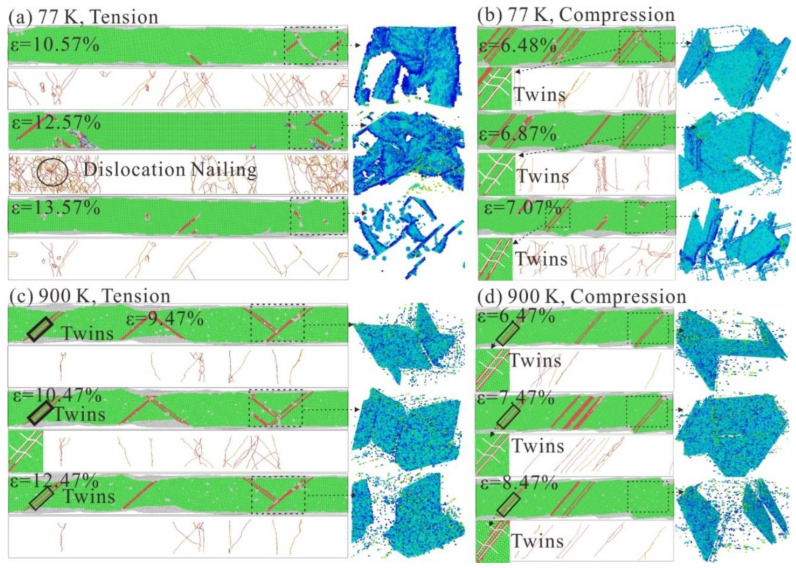
Microstructural evolution of FeNiCrCoCu HEAs with unequal atomic ratio under tensile and compressive loads at different temperatures. (**a**) can, DXA and dislocation microstructure diagram at 77 K with strain of 10.57%, 12.57% and 13.57%; (**b**) canCNA, DXA and dislocation microstructure diagram at 77 K with compressive strain of 6.48%, 6.87% and 7.07%; (**c**) CNA, DXA and dislocation microstructure diagram at 900 K with strain of 9.47%, 10.47% and 12.47%; (**d**) CNA, DXA and dislocation microstructure diagram at 900 K with compressive strain of 6.47%, 7.47% and 8.47%.

Dislocation nails of HEA impede dislocation slip and cause increased stress when the strain is 10.57% (77K), as seen in Figure 6a. When the strain reaches 12.57%, a large number of dislocation nails increase sharply, resulting in peak stress. However, a large amount of dislocation slip results in stress relief when the strain is 13.57%. This is precisely the reason for the sharp drop in stress. As seen in Figure 6c, the number of dislocation nails in HEAs is significantly reduced at 900 K. When the strain is 10.47%, the dislocation nails of HEAs are significantly smaller than the dislocation nails at 77K. In addition, the number of dislocation nails slowly changes around the stress limit at 900K, and the number of sliding surfaces is small. Therefore, its mechanical properties deteriorate significantly when stretched at high temperatures.

HEA has more twins and the dislocations are pinned when the strain is 6.48%, as seen in Figure 6b, resulting in increased stress. The stress reaches its maximum when the strain is 6.87%; this is caused by several dislocation nail points impeding the movement of the dislocation. Unlike stretching, the direction of movement of the slip surface during compression is to squeeze inward at the dislocation nail, and the slip resistance is large. This results in a compressive strength that is higher than the tensile strength. When the strain increases to 7.07%, the sliding surface gradually fails. Dislocation nails begin to disperse and break, resulting in rapid stress release. Therefore, the stress values in the compressive stress-strain curve tend to decrease sharply after the maximum value. As can be seen in Figure 6d, the staggered motion of the high temperature sliding surface forms a complex dislocation network that can prevent dislocation slip more effectively. Therefore, the compressive strength at high temperatures is higher than the compressive strength at low temperatures. When the stress reaches its maximum, the dislocation begins to slide, and the stress is released. However, due to the presence of some twins inside the HEA, the reduction in compressive strength at 900 K is minimal. Therefore, the tension-compression asymmetry at 77 K is attributed to the degree of dislocation nailing and the dislocation slip speed. The tension-compression asymmetry at 900 K is primarily attributed to the formation of dislocation nets and the reduction of twins.

### 3.5. Influence of Element Content on Tension-Compression Asymmetry and Microstructural Evolution Mechanism

**Figure 7:**
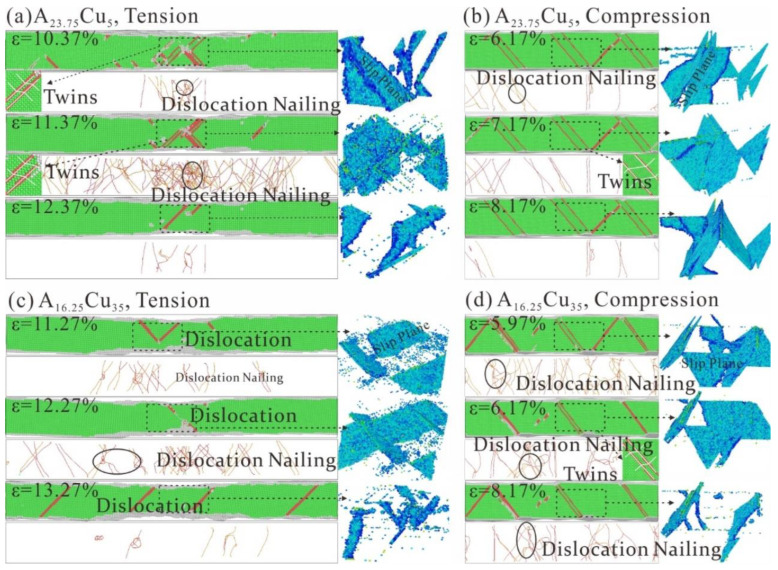
Microstructural evolution of FeNiCrCoCu HEAs under tensile and compression at 300 K. (**a**) CSP, DXA and CAN at tensile A_x_Cu_5_ strains of 10.37%, 11.37% and 12.37%, respectively. (**b**) CSP, DXA and CAN at compressive A*_x_*Cu_5_ at strain 6.17%, 7.17% and 8.17%, respectively. (**c**) CSP, DXA and CAN at tensile B*_y_*Cu_35_ at strain 11.27%, 12.27% and 13.27%, respectively. (**d**) CSP, DXA and CAN at compressive B*_y_*Cu_35_ at strain 5.97%, 6.17%, 8.17%, respectively. A*_x_* is Fe_x_Ni*_x_*Co*_x_*Cr*_x_* (*x* = 23.75, 16.25) and B*_y_* is Ni*_y_*Co*_y_*Cr*_y_*Cu*_y_* (*y* = 23.75, 16.25).

The number of dislocation nails in the HEA decreases as the Cu content increases, resulting in a decrease in tensile strength, as seen in Figure 7a,c. A significant amount of dislocation slip occurs in HEAs with different component proportions, which leads to a sharp drop in stress after the stress reaches its maximum. As can be seen in Figure 7b,d, the twins occur inside the HEAs during compression, which hinder dislocation slip. The compressive strength of HEAs is stronger than tensile strength. When the Cu content increases, the strength decreases due to the small number and size of twins. The increase in Cu content reduces the tension-compression asymmetry of HEAs; this is attributed to a reduction in the number of dislocations nailing and the number and region of twins in HEAs.

### 3.6. Tension-Compression Asymmetry of Polycrystalline HEA and Microscopic Mechanism

To further reveal the difference of mechanical properties between polycrystalline and single crystal HEAs, the tensile and compressive simulations of polycrystalline HEAs with different grain sizes were carried out at 300 K and 900 K. The microscopic mechanism of polycrystalline HEAs under tension and compression load is revealed by analyzing stress-strain curve and microstructure evolution behavior. To eliminate size effects, the tensile and compression model size of polycrystalline HEA is consistent with that of single crystal HEAs, as seen in Table 2.

**Figure 8:**
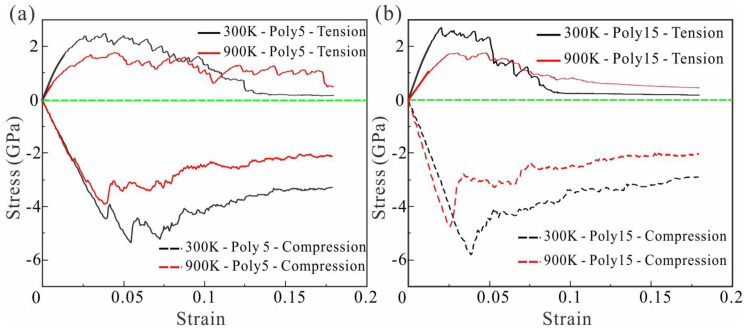
(**a**) Polycrystalline HEA with grain number of 5; (**b**) Stress-strain curves of polycrystalline HEA with grain number of 15 under tensile and compressive loads at 300 K and 900 K, respectively. (The green line indicates that the stress is 0 to distinguish between tension and compression).

As can be seen in Figure 8**,** the tensile and compressive strength of polycrystalline HEA is significantly lower than that of single crystal HEA at 300 K and 900 K. According to Table 4, the tensile and compressive strength of polycrystalline HEA with a grain number of 15 decreased by 31.33% and 30.03%, respectively, at 300 K. Tensile and compressive strength decreased by 34.26% and 53.94%, respectively, at 900 K. However, tensile and compressive strength of polycrystalline HEA with grain number of 5 decreased by 24.78% and 25%, respectively, at 300 K. Moreover, tensile and compressive strength decreased by 27.09% and 45.48%, respectively, at 900 K. Unlike single crystal HEA, polycrystalline HEAs do not have a sharp drop process of stress after reaching tension or compression limit. After polycrystalline HEA reaches the tension or compression limit at 900 K, the stress drop rate is less than that at 300 K. Meanwhile, the finer the grain size of polycrystalline HEA, the slower the rate of stress reduction.

**Figure 9:**
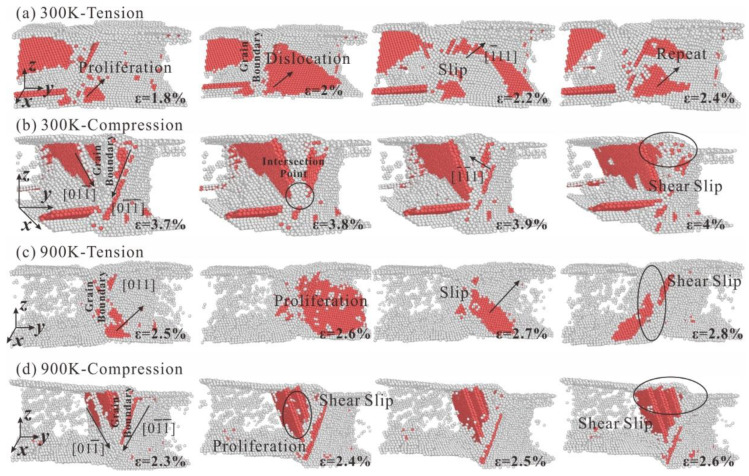
Microstructural evolution of polycrystalline HEA with grain number of 5, (**a**) Tensile load at 300 K, (**b**) Compressive load at 300 K, (**c**) Tensile load at 900 K, and (**d**) Compressive load at 900 K.

To further reveal the different microstructural evolution mechanism of polycrystalline HEA from single crystal HEA, the microstructural evolution of HEA at different temperatures and with different grain size during tension and compression is analyzed. In Figure 9, the microstructural evolution of polycrystalline HEA with a grain number of 5 under tension and compression at 300 K and 900 K can be seen. The red atoms represent dislocation and stacking fault parts, and gray atoms represent grain boundaries and disordered structures. The dislocation slip system in polycrystalline HEA moves at grain boundary and finally forms shear failure. Dislocations begin to multiply below grain boundary and along the $\left[11\bar{1}\right]$ slip, as seen in Figure 9a. When the strain becomes 2.2%, the dislocations in the HEA begin to slip and the stress gradually decreases. However, the stress of the HEA is slowly reduced due to the hindrance of grain boundaries during dislocation slip. The number of dislocations generated in polycrystalline HEA at 900 K is significantly less than that of at 300 K, as seen in Figure 9c. Many free disordered atomic clusters appear due to the increase in temperature, which leads to a decrease in dislocation slip resistance along the [011] direction and a decrease in HEA strength. As can be seen in Figure 9b,d, the dislocations in the HEA grains slip along the $\left[01\bar{1}\right]$ and $\left[0\bar{1}\bar{1}\right]\;$directions during compression. Dislocations are squeezed at grain boundaries, resulting in greater dislocation slip resistance and greater compressive strength than tensile strength. Finally, when the strains are 4% and 2.6%, respectively, HEA has significant shear deformation at the grain boundaries.

**Figure 10:**
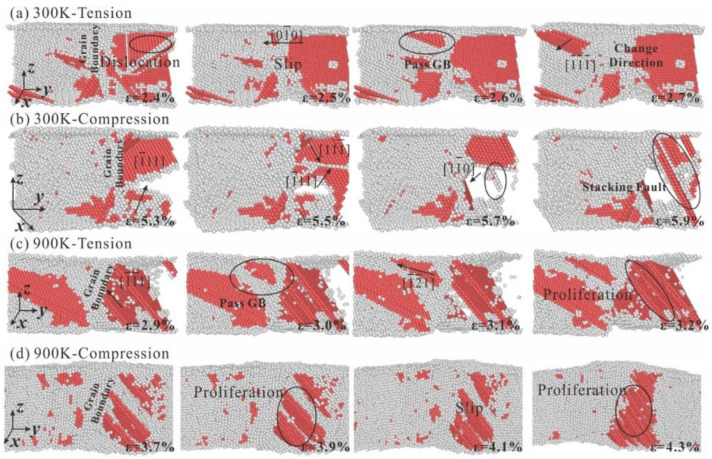
Microstructural evolution of polycrystalline HEA with grain number of 15, (**a**) Tensile load at 300 K, (**b**) Compressive load at 300 K, (**c**) Tensile load at 900 K, and (**d**) Compressive load at 900 K.

The internal microstructure evolution diagram of polycrystalline HEA with a grain count of 15 when it is stretched and compressed at 300 K and 900 K can be seen in Figure 10. Similar to polycrystalline HEA with a grain count of 5, it also produces shear failure at the grain boundary as the number of grains increases. The grain size of polycrystalline HEA decreases with increase of grain number, which results in great changes in the mechanical properties of HEA. As can be seen in Figure 10a, dislocations in grains slip along the $\left[010\right]$ direction at 300 K, and dislocations slip across the grain boundary into another grain and slide along the $\left[\bar{1}\bar{1}\bar{1}\right]$ direction when the strain is 2.6%. As can be seen in Figure 10c, the slip direction changes from $\left[\bar{1}\bar{1}\bar{1}\right]$ to $\left[\bar{1}\bar{2}\bar{1}\right]$ when dislocation crosses the grain boundary at 900 K.

The shift of the slip direction leads to the dislocation entanglement in the slip direction after the dislocation crosses the grain boundary, and the degree of dislocation entanglement increases. Therefore, the higher tensile strength of polycrystalline HEA with smaller grain size is attributed to the increase of resistance and dislocation entanglement when dislocation crosses the grain boundary. In addition, the degree of dislocation entanglement in the grains of the HEA at 900 K is significantly lower than that of at 300 K; the tensile strength of the polycrystalline HEA at 900 K is lower than that at 300 K. The dislocation compresses with each other along directions of $\left[\bar{1}11\right]$ and $\left[11\bar{1}\right]$ at 300 K under compression load, resulting in the increasing stress. When the strain is 5.7%, dislocation nucleation propagates along the $\left[1\bar{1}0\right]$ direction are produced because the stress concentration in the grain reaches a certain degree. When the strain is 5.9%, the dislocation nucleation grows and many dislocation entanglements occur, which leads to the further increase of stress. Dislocations in the grain boundary at 900 K proliferate continuously to produce dislocations sliding along the $\left[\bar{1}\bar{1}1\right]$ direction in Figure 10d. When the strain is 4.3%, the degree of dislocation entanglement is the largest.

### 3.7. Influence and Mechanism of HEA Coating on Mechanical Properties

**Figure 11:**
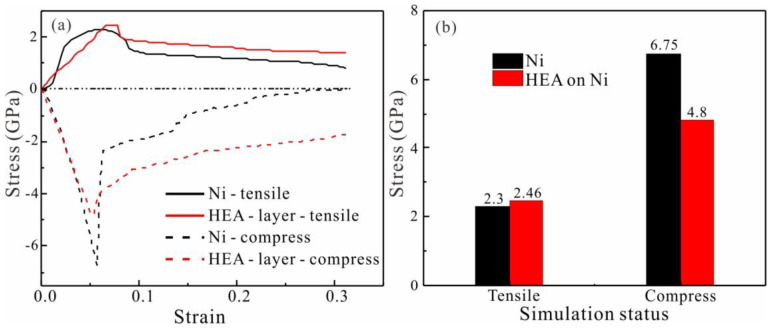
(**a**) Stress-strain curve of pure Ni and Ni reinforced with the HEAs coating at 300 K; (**b**) Histogram of tensile and compressive strength of Fe_33_Ni_32_Cr_11_Co_11_Cu_13_ HEA coating and pure Ni.

The stress-strain curves of pure Ni and Ni reinforced with Fe_33_Ni_32_Cr_11_Co_11_Cu_13_ HEAs under tensile and compressive load are shown in Figure 11a. Both samples had the same overall size of 17.8 × 53.4× 7.12 nm^3^. The thickness ratio of HEA and Ni in the coating reinforced sample is one to three. As can be seen in Figure 11b, the tensile strength of HEA coated reinforced Ni increased from 2.3 GPa to 2.46 GPa, while the compressive strength decreased from 6.75 GPa to 4.8 GPa. However, the compressive strength of both samples was higher than the tensile strength. We found that the tensile-compression asymmetry of Ni reached 65.97% (Table 5). The HEA coating reinforced Ni content is reduced to 48.8%. The results show that the addition of HEA changes the tension-compression asymmetry of the Ni matrix.

**Figure 12:**
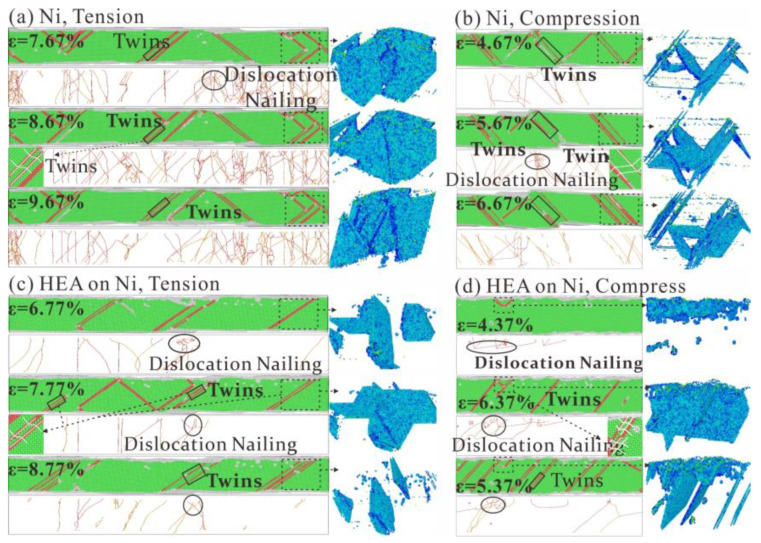
Dislocation analysis diagram obtained by tensile-compression of pure Ni and Ni reinforced with the Fe_33_Ni_32_Cr_11_Co_11_Cu_13_ HEAs coating at 300 K. (**a**) CSP, DXA and CAN at tensile (pure Ni) strain of 7.67%, 8.67% and 9.67%, respectively. (**b**) CSP, DXA and CAN at compressive (pure Ni) strain of 4.67%, 5.67% and 6.67%, respectively. (**c**) CSP, DXA and CAN at tensile (Ni with the HEAs coating) strain of 6.77%, 7.77% and 8.77%, respectively. (**d**) CSP, DXA and CAN at compressive (pure Ni) strain of 4.37%, 5.37% and 6.37%, respectively.

We further analyzed the microstructural evolution behavior of the two models. Dislocation nails are generated inside pure Ni when strain reaches 7.67% at 300K, as seen in Figure 12a. Dislocation slipping of the HEA are entangled with each other when strain increases to 8.67%. As the strain increases, a large amount of dislocation slip leads to a decrease in stress. As seen in Figure 12b, the compression of pure Ni produces more twins internally, while the dislocation slip is limited, resulting in the compressive strength of pure Ni being greater than tensile strength. In addition, many dislocations slip at strain 5.67%, and the stress begins to drop sharply after reaching the compression limit.

The model with the HEAs coating showed more twinning, as seen in Figure 12c, resulting in an increase in its tensile strength. The microscopic mechanism is that dislocation inside the HEAs coating climbs to Ni, forming a large slip surface and promoting generation of twins. This is due to different atomic sizes of the principal element of the HEA, which leads to obstruction of dislocation slip process; stress concentration promotes the generation of twinning. As seen in Figure 12d, dislocations in the HEAs during compression climb into Ni and promotes generation of twins. However, the twins in Ni degrade faster at the HEA coating, resulting in a decrease in compressive strength of Ni. Therefore, the climbing of dislocations in HEAs promotes generation of twins in Ni, affecting tensile and compressive mechanical properties of Ni. The HEA coating enhances tensile strength of Ni, reduces compressive strength of Ni and reduces the tension-compression asymmetry of Ni matrix.

Models coated with HEAs showed more twinning under tensile loading, as seen in Figure 12c, resulting in an increase in their strength. The microscopic mechanism is that the dislocation within the HEAs coating climbs towards Ni, forming a large slip surface and promoting the generation of twins. This is due to the different atomic sizes of the primary elements of HEA, which leads to the obstruction of the dislocation-slip process and the stress concentration to promote the production of twins. As seen in Figure 12d, dislocations in HEA during compression are pressed into Ni and promote twin formation. However, the twins in Ni degrade faster at the HEA coating, resulting in a decrease in the compressive strength of the Ni. Thus, the dislocations’ climb in HEA promotes the formation of twins in Ni, which affects the tensile and compressive mechanical properties of Ni. It is found that HEA coating can improve the tensile strength of nickel, but it weakens the compressive strength of Ni. The beneficial changes of the two strengths reduce the tension-compression asymmetry of the Ni matrix.

## 4. Conclusions

In this paper, the mechanical properties of FeNiCrCoCu HEAs with unequal atomic ratio are studied by combining ML and MD. Among the three ML algorithms (MLR, BPNN, RF) adopted, the MLR model performed well in predicting tensile strength of FeNiCrCoCu HEAs. This is attributed to strong linear correlation between tensile strength of the HEAs and the content of principal elements. Mechanical properties of FeNiCrCoCu HEAs with unequal atomic ratio were predicted using the optimal MLR model, and tensile strength of Fe_33_Ni_32_Cr_11_Co_11_Cu_13_ HEAs (HEAs with optimum mechanical properties) reached 28.25 GPa. ML results were verified using MD, and it was found that error between predicted tensile strength and the simulated was within 0.5%.

The FeNiCrCoCu HEAs were simulated for tension-compression at different temperatures and composition ratios. We found that the influence of temperature on the tensile and compressive mechanical properties of FeNiCrCoCu HEAs is greater than the influence of the content of the primary element. The generation of twins and the small degree of destruction of the sliding surface result in the compressive strength of HEA being higher than the tensile strength. In addition, temperature affects the twins more than the elements, resulting in a greater influence of temperature on the tensile-compression asymmetry of HEA. The mechanical properties of polycrystalline HEA are significantly lower than that of single crystal HEA. In addition, the tensile and compression property of polycrystalline HEA with larger particle sizes is not as good as HEA with smaller particle sizes. This is attributed to the obstruction of grain boundaries and the degree of dislocation entanglement when dislocations cross grain boundaries in polycrystalline HEAs. In addition, due to the presence of grain boundaries, the stress of polycrystalline HEAs does not decrease rapidly after reaching tensile and compression limits. Finally, at 300 K, tensile and compressive loads are applied to pure nickel and nickel coated with FeNiCrCoCu HEAs. The results show that the microscopic mechanism of HEAs strengthening nickel is that the climb of dislocations in HEAs promotes the formation of twins in nickel, thereby improving the strength of nickel. After HEAs coating strengthening, the tensile-compression asymmetry of nickel was reduced by 18%. This is due to twin degradation due to the different atomic sizes of the main elements of HEA.

## Figures and Tables

**Table 1:** Parameters in EAM potential functions [37].

Parameter	Value
Get points of ρ ($N_{\rho })$	2000
Picking interval of $\;\rho \;\left(\Delta \rho \right)$	$1\times 10^{-3}$ Å
Get points of r	3000
Picking interval of r (Δr)	$1\times 10^{-3}$ Å
Truncation distance of r (*rcut)*	5.804 Å

**Table 2:** Proportion and size of FeNiCrCoCu HEAs with unequal atomic ratio.

Component Proportion of HEA	Size of HEA
Fe_5_Ni_23.75_Cr_23.75_Co_23.75_Cu_23.75_	17.8 nm×53.4 nm×7.12 nm
Fe_20_Ni_20_Cr_20_Co_20_Cu_20_	
Fe_35_Ni_16.25_Cr_16.25_Co_16.25_Cu_16.25_	
Fe_23.75_Ni_23.75_Cr_23.75_Co_23.75_Cu_5_	
Fe_20_Ni_20_Cr_20_Co_20_Cu_20_	
Fe_16.25_Ni_16.25_Cr_16.25_Co_16.25_Cu_35_	

**Table 3:** Tension-compression asymmetry of HEAs at different temperatures and component proportions, where A*_x_* is Fe_x_Ni*_x_*Co*_x_*Cr*_x_* (*x* = 23.75, 20, 16.25) and B*_y_* is Ni*_y_*Co*_y_*Cr*_y_*Cu*_y_* (*y* = 23.75, 16.25).

Influence Factor	Variable Value	Tensile Strength (GPa)	Compress Strength (GPa)	Asymmetry Ratio (%)
Temperature	77 K	4.29	7.21	40.5
	300 K	3.51	7.76	54.81
	600 K	2.88	11.05	73.94
	900 K	2.51	8.51	70.53
Element Content	A_23.75_Cu_5_	3.19	7.01	54.5
	A_20_Cu_20_	3.2	7.52	57.45
	A_16.25_Cu_35_	3.28	6.53	49.77
	B_23.75_Fe_5_	3.1	10.91	71.59
	B_16.25_Fe_35_	3.32	6.56	49.39

**Table 4:** Tensile strength, compressive strength and tension-compression asymmetry of polycrystalline and single crystal HEAs at 300 K and 900 K.

Matrix	Temperature	Tensile Strength (GPa)	Compress Strength (GPa)	Asymmetry Ratio (%)
Crystal HEA	300 K	3.51	7.76	54.81
	900 K	2.51	8.51	70.53
Polycrystalline HEA (5 Grains)	300 K	2.41	5.43	55.62
	900 K	1.65	3.92	57.91
Polycrystalline HEA (15 Grains)	300 K	2.64	5.82	54.64
	900 K	1.83	4.64	60.56

**Table 5:** Tensile strength, compressive strength and asymmetry ratio of pure Ni and Ni reinforced with the HEAs coating.

Influence Factor	Tensile Strength (GPa)	Compress Strength (GPa)	Asymmetry Ratio (%)
Ni	2.3	6.75	65.93
HEA Coating	2.46	4.81	48.85

## Data Availability

Because private data is not available, please contact us if necessary.

## References

[1] Jiang L., Lu Y., Dong Y., Wang T., Cao Z., Li T. (2014). Annealing effects on the microstructure and properties of bulk high-entropy CoCrFeNiTi0.5 alloy casting ingot. Intermetallics.

[2] He J.Y., Wang H., Huang H.L., Xu X.D., Chen M.W., Wu Y., Liu X.J., Nieh T.G., An K., Lu Z.P. (2016). A precipitation-hardened high-entropy alloy with outstanding tensile properties. Acta Mater..

[3] Fu Z., Chen W., Wen H., Zhang D., Chen Z., Zheng B., Zhou Y., Lavernia E.J. (2016). Microstructure and strengthening mechanisms in an FCC structured single-phase nanocrystalline Co25Ni25Fe25Al7.5Cu17.5 high-entropy alloy. Acta Mater..

[4] Braic V., Balaceanu M., Braic M., Vladescu A., Panseri S., Russo A. (2012). Characterization of multi-principal-element (TiZrNbHfTa)N and (TiZrNbHfTa)C coatings for biomedical applications. J. Mech. Behav. Biomed..

[5] Chang S.-Y., Li C.-E., Huang Y.-C., Hsu H.-F., Yeh J.-W., Lin S.-J. (2014). Structural and Thermodynamic Factors of Suppressed Interdiffusion Kinetics in Multi-component High-entropy Materials. Sci. Rep..

[6] Yeh A.-C., Chang Y.-J., Tsai C.-W., Wang Y.-C., Yeh J.-W., Kuo C.-M. (2014). On the Solidification and Phase Stability of a Co-Cr-Fe-Ni-Ti High-Entropy Alloy. Metall. Mater. Trans. A.

[7] Maiti S., Steurer W. (2016). Structural-disorder and its effect on mechanical properties in single-phase TaNbHfZr high-entropy alloy. Acta Mater..

[8] Tang Z., Yuan T., Tsai C.-W., Yeh J.-W., Lundin C.D., Liaw P.K. (2015). Fatigue behavior of a wrought Al0.5CoCrCuFeNi two-phase high-entropy alloy. Acta Mater..

[9] Licavoli J.J., Gao M.C., Sears J.S., Jablonski P.D., Hawk J.A. (2015). Microstructure and Mechanical Behavior of High-Entropy Alloys. J. Mater. Eng. Perform..

[10] Hemphill M.A., Yuan T., Wang G.Y., Yeh J.W., Tsai C.W., Chuang A., Liaw P.K. (2012). Fatigue behavior of Al0.5CoCrCuFeNi high entropy alloys. Acta Mater..

[11] Thurston K.V.S., Gludovatz B., Hohenwarter A., Laplanche G., George E.P., Ritchie R.O. (2017). Effect of temperature on the fatigue-crack growth behavior of the high-entropy alloy CrMnFeCoNi. Intermetallics.

[12] Seifi M., Li D., Yong Z., Liaw P.K., Lewandowski J.J. (2015). Fracture Toughness and Fatigue Crack Growth Behavior of As-Cast High-Entropy Alloys. JOM.

[13] Senkov O.N., Scott J.M., Senkova S.V., Miracle D.B., Woodward C.F. (2011). Microstructure and room temperature properties of a high-entropy TaNbHfZrTi alloy. J. Alloys Compd..

[14] Cao T., Guo W., Lu W., Xue Y., Lu W., Su J., Liebscher C.H., Li C., Dehm G. (2022). Strain rate dependent deformation behavior of BCC-structured Ti29Zr24Nb23Hf24 high entropy alloy at elevated temperatures. J. Alloys Compd..

[15] Tsai C.-W., Tsai M.-H., Yeh J.-W., Yang C.-C. (2010). Effect of temperature on mechanical properties of Al0.5CoCrCuFeNi wrought alloy. J. Alloys Compd..

[16] Sun W., Huang X., Luo A.A. (2017). Phase formations in low density high entropy alloys. Calphad.

[17] Wu Y.D., Cai Y.H., Wang T., Si J.J., Zhu J., Wang Y.D., Hui X.D. (2014). A refractory Hf25Nb25Ti25Zr25 high-entropy alloy with excellent structural stability and tensile properties. Mater. Lett..

[18] Li Z., Pradeep K.G., Deng Y., Raabe D., Tasan C.C. (2016). Metastable high-entropy dual-phase alloys overcome the strength–ductility trade-off. Nature.

[19] Huang H., Wu Y., He J., Wang H., Liu X., An K., Wu W., Lu Z. (2017). Phase-Transformation Ductilization of Brittle High-Entropy Alloys via Metastability Engineering. Adv. Mater..

[20] Zhang Y., Zuo T.T., Tang Z., Gao M.C., Dahmen K.A., Liaw P.K., Lu Z.P. (2014). Microstructures and properties of high-entropy alloys. Prog. Mater Sci..

[21] Palguna Y., Kotla S., Korla R. (2023). High temperature deformation behavior of Al0.2CoCrFeNiMo0.5 high entropy alloy: Dynamic strain ageing. J. Alloys Compd..

[22] Brechtl J., Feng R., Liaw P.K., Beausir B., Jaber H., Lebedkina T., Lebyodkin M. (2023). Mesoscopic-scale complexity in macroscopically-uniform plastic flow of an Al0.3CoCrFeNi high-entropy alloy. Acta Mater..

[23] Tirunilai A.S., Hanemann T., Weiss K.P., Freudenberger J., Heilmaier M., Kauffmann A. (2020). Dislocation-based serrated plastic flow of high entropy alloys at cryogenic temperatures. Acta Mater..

[24] Xie L., Brault P., Thomann A.-L., Bauchire J.-M. (2013). AlCoCrCuFeNi high entropy alloy cluster growth and annealing on silicon: A classical molecular dynamics simulation study. Appl. Surf. Sci..

[25] Tian Y., Fang Q., Li J. (2020). Molecular dynamics simulations for nanoindentation response of nanotwinned FeNiCrCoCu high entropy alloy. Nanotechnology.

[26] Qi Y., Chen X., Feng M. (2020). Molecular dynamics-based analysis of the effect of temperature and strain rate on deformation of nanocrystalline CoCrFeMnNi high-entropy alloy. Appl. Phys. A-Mater..

[27] Brun F., Yoshida T., Robson J.D., Narayan V., Bhadeshia H.K.D.H., MacKay D.J.C. (1999). Theoretical design of ferritic creep resistant steels using neural network, kinetic, and thermodynamic models. Mater. Sci. Technol..

[28] Conduit B.D., Jones N.G., Stone H.J., Conduit G.J. (2018). Probabilistic design of a molybdenum-base alloy using a neural network. Scripta Mater..

[29] Faizabadi M.J., Khalaj G., Pouraliakbar H., Jandaghi M.R. (2014). Predictions of toughness and hardness by using chemical composition and tensile properties in microalloyed line pipe steels. Neural. Comput. Appl..

[30] Zhang L., Qian K., Schuller B.W., Shibuta Y. (2021). Prediction on Mechanical Properties of Non-Equiatomic High-Entropy Alloy by Atomistic Simulation and Machine Learning. Metals.

[31] Li L., Xie B., Fang Q., Li J. (2021). Machine Learning Approach to Design High Entropy Alloys with Heterogeneous Grain Structures. Metall. Mater. Trans. A.

[32] Zheng T., Hu X., He F., Wu Q., Han B., Chen D., Li J., Wang Z., Wang J., Kai J.-J. (2021). Tailoring nanoprecipitates for ultra-strong high-entropy alloys via machine learning and prestrain aging. J. Mater. Sci. Technol..

[33] Bhandari U., Rafi M.R., Zhang C., Yang S. (2021). Yield strength prediction of high-entropy alloys using machine learning. Mater. Today Commun..

[34] Zhang L., Qian K., Huang J., Liu M., Shibuta Y. (2021). Molecular dynamics simulation and machine learning of mechanical response in non-equiatomic FeCrNiCoMn high-entropy alloy. J. Mater. Res. Technol..

[35] Plimpton S. (1995). Fast Parallel Algorithms for Short-Range Molecular Dynamics. J. Comput. Phys..

[36] Li Z., Zhao S., Ritchie R.O., Meyers M.A. (2019). Mechanical properties of high-entropy alloys with emphasis on face-centered cubic alloys. Prog. Mater Sci..

[37] Farkas D., Caro A. (2018). Model interatomic potentials and lattice strain in a high-entropy alloy. J. Mater. Res..

[38] Zhao S. (2021). Role of chemical disorder and local ordering on defect evolution in high-entropy alloys. Phys. Rev. Mater..

[39] Stukowski A. (2010). Visualization and analysis of atomistic simulation data with OVITO–the Open Visualization Tool. Model Simul. Mater. Sci..

[40] Kelchner C.L., Plimpton S.J., Hamilton J.C. (1998). Dislocation nucleation and defect structure during surface indentation. Phys. Rev. B.

[41] Urrutia Bañuelos E., Contreras Aburto C., Maldonado Arce A. (2016). A common neighbor analysis of crystallization kinetics and excess entropy of charged spherical colloids. J. Chem. Phys..

[42] Stukowski A., Andreoni W., Yip S. (2018). Dislocation Analysis Tool for Atomistic Simulations. Handbook of Materials Modeling: Methods: Theory and Modeling.

[43] Burchfield P.B. (1971). Multiple Linear Regression. J. Qual. Technol..

[44] Li J., Cheng J.-H., Shi J.-Y., Huang F. (2012). Brief Introduction of Back Propagation (BP) Neural Network Algorithm and Its Improvement. Advances in Computer Science and information Engineering, Proceedings of the 2012 2nd International Conference on Computer Science and Information Engineering (CSIE2012) May 19-20, Zhengzhou, China.

[45] Biau G., Scornet E. (2016). A random forest guided tour. TEST.

